# Behind the mask: Random and selective masking in transformer models applied to specialized social science texts

**DOI:** 10.1371/journal.pone.0318421

**Published:** 2025-02-21

**Authors:** Joan C Timoneda, Sebastián Vallejo Vera

**Affiliations:** 1 Department of Political Science, Purdue University, West Lafayette, Indiana, United States of America; 2 Department of Political Science, University of Western Ontario, London, Ontario, Canada; The University of Lahore, PAKISTAN

## Abstract

Transformer models such as BERT and RoBERTa are increasingly popular in the social sciences to generate data through supervised text classification. These models can be further trained through Masked Language Modeling (MLM) to increase performance in specialized applications. MLM uses a default masking rate of 15 percent, and few works have investigated how different masking rates may affect performance. Importantly, there are no systematic tests on whether *selectively* masking certain words improves classifier accuracy. In this article, we further train a set of models to classify fake news around the coronavirus pandemic using 15, 25, 40, 60 and 80 percent *random* and *selective* masking. We find that a masking rate of 40 percent, both random and selective, improves within-category performance but has little impact on overall performance. This finding has important implications for scholars looking to build BERT and RoBERTa classifiers, especially those where one specific category is more relevant to their research.

## Introduction

**Fig 1:**
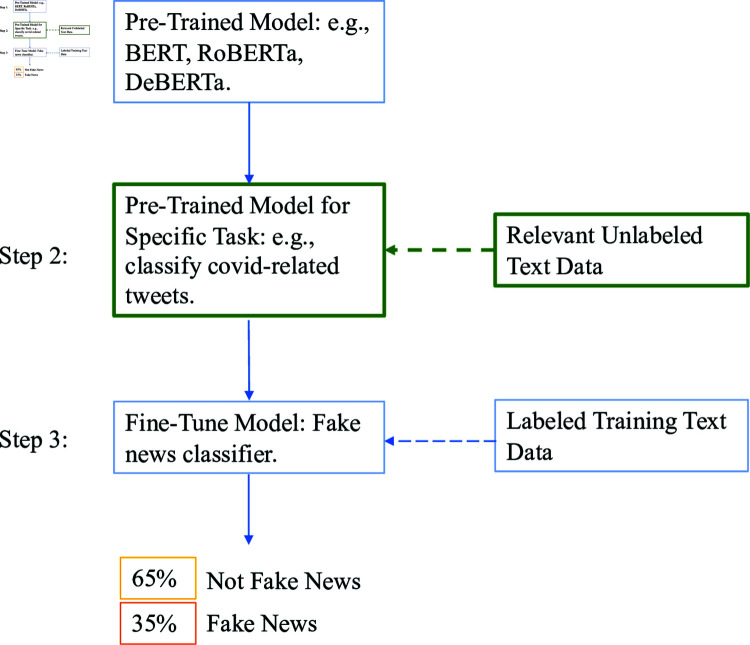
Representation of a downstream classification task using transformer models. In step 1, researchers choose a pretrained Transformers model (e.g., BERT, RoBERTa). In step 2, researchers *further* train the model of choice with additional relevant unlabeled text. In step 3, researchers fine-tune the pretrained model for a specific classification task. While step 2 is optional, in previous work we show that further pretraining a model can lead to improvements in performance [3].

For supervised text classification tasks, a suite of open-source Transformers models has become increasingly popular in the social sciences: BERT and RoBERTa. Transformers are a deep-learning neural network architecture that transforms an input sequence (to encode) into an output sequence (to decode). The encoder-decoder architecture of these models makes them especially powerful for supervised classification, a task in which other well-known alternatives such as OpenAI’s GPT-3.5 and GPT-4 may perform similarly [1, 2] but are not open-source. The open-source nature and flexibility of BERT and RoBERTa allows researchers to adapt these models to specialized texts in English and cross-lingual applications [3] across domains from sociology and political science [4, 5] to biology and medicine [6–11]. Fig 1 describes the process whereby applied researchers can further train and fine-tune Transformer models. To learn, the BERT family of models use Masked Language Modeling (MLM): some percentage of words in the training data are masked, and the model then learns to predict the masked words. By default, masking applies randomly to 15% of the words in the training text. This raises two questions. First, does performance vary with a higher rate of masking? Second, what if we *selectively* mask certain words that are more meaningful than others to the task at hand?

This article addresses these two questions within the scope of supervised text classification. We focus on BERT and RoBERTa, the former due to its popularity and the latter to its higher level of performance [3]. The intuition is that performance will vary significantly with different masking strategies. First, higher masking levels should increase downstream performance in classification tasks, as the model is forced to understand more tokens in context. This, however, has diminishing returns, as masking too many tokens hinders performance. Specifically, we find that masking more than 15% of tokens improves category-specific performance, but the best masking rate depends on the task. We recommend that researchers test multiple masking rates to maximize category-specific performance. Second, selective masking, or the targeted masking of specialized and high-information words during pretraining, should improve performance. Note that high-information words are tokens that are more likely to appear when labelling certain categories. For example, when labelling emotions, “happy” might be a high-information word for positive emotion, and “sad” might be a high-information word for negative emotion. As the name suggests, these words provide high information to the model about the nature of the text. We expect that asking the model to predict specific words more often during pretraining should result in better contextual understanding when building a classifier (fine-tuning). We find that selective masking of 25 and 40 percent improves category-specific performance. At 15 percent, random masking performs better than selective masking. Lastly, we expect lower performance with masking rates over 60 percent, as the model has too little unmasked data to learn effectively.

As the application of Transformers grows in scope, domain-specific models have proliferated [2, 12]. However, most of these endeavors have focused primarily on leveraging domain-specific pretraining data. Our work contributes to the growing applied literature on Transformer models, adding new insights into the process of *selective masking*. We show that performance increases with higher masking rates, and that gains are especially strong in category-specific performance. It also resonates with computer science literature that has started to explore masking in Transformer models [13–15]. The novelty of our argument lies in (1) applying *selective* masking to highly nuanced, niche social science text and (2) the emphasis on category-based performance as opposed to overall model performance. The reason for the latter is that scholars often generate data for specific categories, not *all* categories that a model classifies. By further training models with higher levels of masking, applied researchers can create better data that will have fewer biases in downstream tasks [16].

This article makes four contributions. First, we provide new evidence that increasing the random masking rate in RoBERTa models in social science texts increases category-based performance. Second, we show that selectively masking between 25 and 40 percent of tokens can significantly improve classifier performance. Third, our findings are especially relevant for highly specialized texts, where technical language or the emergence of new terms are central to contextual understanding. Fourth, this article also has major implications with research using rare events data, as selective masking can improve performance for categories with lower frequencies.

## What is masking and why 15% of tokens is not enough

**Fig 2:**
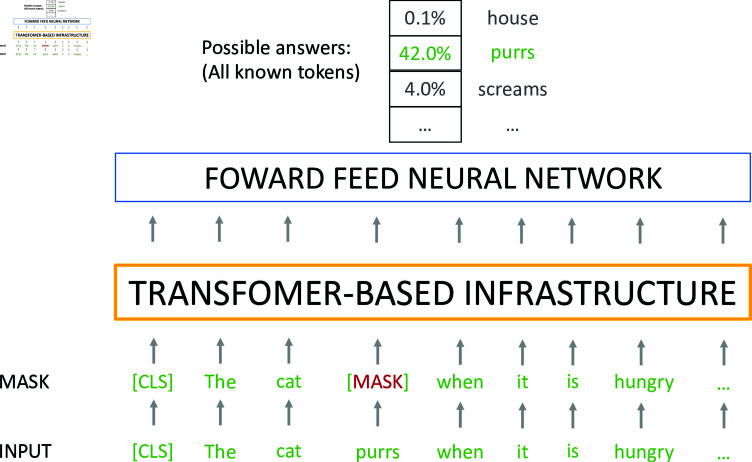
Representation of the masked language modeling. Transformer models randomly mask 15% of tokens. After running the corpus through the Transformers and neural-network architecture, it asks the model to predict the masked word.

In previous work, we provide an overview of Transformers-based models and explain how MLM fits in the Transformers infrastructure [3]. During pretraining, MLM masks a proportion of tokens and induces the model to apply its self-attention mechanism to predict the masked token. For instance, in the sentence ‘I love visiting the windy city,  < MASK > , the cultural and commercial capital of the Midwest’, BERT and RoBERTa will use the information before and after  < MASK >  to predict ‘Chicago’. While ‘windy city’ may provide a clue, the fact that the city is an important Midwest metropolis is key in predicting the word correctly. These models use all relevant information in the sentence (‘city’, ‘windy’, ‘midwest’, ‘metropolis’, and ‘commercial capital’) to come up with a probability for the most likely candidate token to replace the  < MASK > . During pretraining, BERT and similar models use MLM to predict a set percentage of all tokens in the unlabelled training data. By default, 15% of tokens are randomly selected, masked, and predicted. Fig 2 provides a graphical description of MLM.

Yet the rationale behind the decision to mask 15% of tokens remains unclear. This practice has been the norm since [17] adopted it in their foundational BERT paper. The authors argued that excessive masking can hinder a model’s ability to learn effective representations, while insufficient masking can result in inefficient training. However, the choice of 15% is arbitrary and there is a lack of rigorous empirical validation regarding its impact on performance. Even newer models after BERT, such as RoBERTa and DeBERTa, use the same 15% masking rate without thorough reasoning [18–20]. Note that while RoBERTa and DeBERTa randomly mask tokens, they employ a dynamic masking strategy, where the masking is implemented anew in each epoch, changing the masked tokens in each training sequence. However, dynamic masking does not increase performance when compared to static masking and, while it has some efficiency benefits [18], it also increases the computational costs [21].

Extant work shows that different masking rates and masking strategies can lead to improvements in *overall* model performance. [13] find that increasing the masking rate can improve overall model performance in pretraining LLMs. For GLUE and SQuAD, two common benchmark resources used to evaluate natural language understanding systems, masking 40% of tokens using BERT increases F1 performance metrics by 2 percentage points. [22] find that this effect is linked to position encoding. The higher the masking rate, the more important for the model to know the position of each token in a sentence. Research has also explored the effect of time-variant masking, i.e. increasing the masking rate at different layers or steps of the process [15, 23, 24]. Results suggest only modest performance increases, but considerable gains in efficiency in terms of computational time (compared to time-invariant MLM). Following a similar logic, [25] propose *demasking* tokens at different rates. They show that the gradual relaxation of attention mask in BERTS models leads to gains in performance. We refer to tests on the overall masking rates as ‘general masking’. No work, to our knowledge, has explored the effect of masking on category-specific performance. With more researchers using BERT and RoBERTa models for measurement, it is important to know how to maximize performance within categories, not just overall.

Another line of research on masking strategies focuses on *which* words are being masked. PMI-masking, for example, identifies n-grams with high collocation values–the co-occurrence of series of tokens at levels much greater than would be predicted simply by their individual frequencies in the corpus–and increases the probability of having these words masked during pretraining [26]. Similarly, [27] utilize selective masking to improve few-shot text classification by filtering out irrelevant information. PMI-masking is more time-efficient during pretraining, and maintains model performance using less data. Alternatively, KeyWord Masking (KWM) uses topic-specific keyword dictionaries to select highly informative tokens to mask and have the model conduct name entity recognition (NER) task during pretraining [14]. They find that KWM outperforms traditional methods in restoring domain-specific entities. We refer to the masking of targeted, specific tokens as ‘selective masking’ (see also [28]). An alternative strategy has been employed by [29], who selectively mask tokens at the fine-tuning stage and achieve performance gains in reasoning tasks (e.g., GSM8K).

This work complements and builds on current state-of-the-art research in social science using word embeddings and deep learning. [30] and [5] have developed relevant, accurate tools to improve context-specific machine understanding of text through specialized word embeddings. Other work has made important contributions to our understanding of topic models in social science applications [31, 32]. Our approach provides an alternative view of supervised classification using deep learning LLMs, especially in contexts where text is highly nuanced and specialized [3].

## Data and analysis

We test both (1) general masking rates and (2) selective masking using one social science application: detecting fake news around the COVID-19 pandemic [33, 34], an important source of data in various studies on ideology, misinformation, and democratic backsliding. As shown in Fig 1, the downstream process to train a classification model requires researchers 1) to choose a pretrained Transformers model, 2) further train it with additional unlabeled text, and 3) fine-tuning the resulting model for a specific classification task. Here, we focus on changing the masking strategy used during step 2 (further training a Transformers model).

To train our classifier, we use a manually labeled dataset of true and fake news around the Covid-19 pandemic (see [35]). The authors of the project gathered 7,179 news headlines and Twitter posts containing the words ‘coronavirus’ or ‘covid’ between December 2019 and September 2020. The dataset is available at: https://github.com/MickeysClubhouse/COVID-19-rumor-dataset. See Appendix B for a detailed description of the COVID-19 rumour dataset and a discussion of its limitations and potential biases. Through fact-checking websites, they labeled each story as fake, true, or undetermined. [36] define ‘fake news’ as ‘fabricated information that mimics news media content in form but not in organizational process or intent’. Examples of fake news from the aforementioned dataset are: ‘Coronavirus was created in a government lab as a bioweapon and then released on the people of China’ or ‘Japanese government doctors advise that taking a few sips of water every 15 mins will prevent the new coronavirus from entering your windpipe and lungs’. The final dataset has 3,681 fake (51.27%), 1,878 true (26.16%), and 1,620 undetermined (22.57%) news stories and tweets. We select 500 from each category for fine-tuning. The mean length for the headlines is 21.56 words, and the longest is 143 words, resulting in an average of 29.75 tokens per sentence using the RoBERTa tokenizer and a maximum number of tokens of 160.

Data on the coronavirus pandemic provides a clear example of the advantages of further training a Transformer model. When RoBERTa was originally trained in 2018-19, Covid-19 did not exist. The model thus cannot know what Covid-19 is or how the words ‘coronavirus’ or ‘covid’ are used in context today. Our solution is to further train a new RoBERTa model with new data containing the words ‘coronavirus’ and ‘covid’ and add those two vocabulary elements to the tokenizer (see [3] for a guide on how to further train a RoBERTa model for this specific task.) While the meaning of these two Covid-related words does not provide information about to the truth of a tweet’s content, it allows the model to understand the sentence better and thus detect new, informative patterns. To help the model distinguish between false and true news, we also add the token ‘CDC’ (Centers for Disease Control in the USA) to the tokenizer. We give the CDC token the mean of the embeddings for ‘authority’ and ‘health’. The new model should be able to classify texts containing these two new words more accurately than original BERT or RoBERTa. Lastly, our choice of topic and data also illustrate the generalizability of our approach in a clear and accessible way. Researchers who specialize in a given subfield should consider adding tokens to the classifier if (1) the model is unlikely to have been asked to predict them in the original MLM step and (2) the underlying words are sufficiently niche but common to their application that they can reasonably impact performance. For this example, we estimate TF-IDF metrics for our training set, and choose the highest value tokens from each category that do not appear on the RoBERTa dictionary.

To further train the model, we used two unlabelled corpora. First are 6,079 abstracts from academic articles on the topic of Covid-19 and the coronavirus pandemic. The data are available at https://www.kaggle.com/datasets/phiitm/covid19-research-preprint-data. Second is 1GB of short news headlines around the Covid-19 pandemic obtained from Twitter in English. We downloaded the data using Twitter’s API v2. We queried English tweets containing the text ’covid’ and ’coronavirus’, between March 30, 2020 and May 1, 2020. In total, our dataset includes 4.8 million unique tweets. Many of these tweets contain the term ‘CDC’, which is the third most common term in true tweets. The format of these texts closely resembles the training data used to fine-tune the classifier (tweets). Importantly, none of the unlabelled tweets used in the further training step are included in the final labelled data for fine-tuning. We will refer to the resulting models trained to recognize Covid-related text as RoBERTa-Covid models.

To test the variation across (1) general and (2) selective masking, we run two different sets of models. First, we further train five RoBERTa-Covid models with fully *random* masking of 15, 25, 40, 60, and 80 percent. We choose these masking rates to lower computational costs while still providing sufficient insight into how different masking rates result in performance changes. This means that, for instance in the fourth model, MLM will mask 60% of all tokens at random and ask RoBERTa to predict them based on the context. We then fine-tune one classifier for each model using the labeled data and evaluate their individual performance. Second, we further train another set of five RoBERTa-Covid models with *selective* masking. In this procedure, we ask MLM to mask the same amount of random words as in general masking *as well as* the same percentage of appearances of two other tokens: ‘covid’ and ‘coronavirus’. The selective masking approach was chosen to evaluate the model’s ability to predict domain-specific tokens related to the Covid-19 pandemic, as these two tokens are likely to be crucial for accurate classification in this domain. For example, in the 40% selective masking model, MLM will mask 40% of all tokens at random *and* 40% of all occurrences of ‘covid’ and ‘coronavirus’. In the 25% selective masking model, it will mask 25% of tokens at random and 25% of ‘covid’ and ‘coronavirus’ tokens, and so on. Mirroring general and selective masking percentages helps us directly compare performance across both categories. All models include an early stopping mechanism to prevent overfitting. Training is stopped when validation performance degrades (training loss declines when compared to the previous epoch.)

In addition to overall performance, we are interested in category-specific gains. In classification tasks, performance across categories will vary, especially in unbalanced dataset. In some cases, important gains in lower accuracy categories, can offset overall F1 scores when accompanied to small loses in highly accurate (and populated) categories. Domain-specific pretraining of Transformer models, as well as masking strategies as the one proposed in this paper, can have positive performance effects on hard to predict categories (see [37, 38]). Furthermore, researchers and practitioners will value Type I and Type II error differently depending of the context of the study, and changes in category-specific performance are of relevance. To address these concerns, we present performance statistics for each target category in our downstream tasks.

## Results

Table 1 displays the main results of the article. It shows the performance differences by random and selective masking strategies across five different models and masking rates. All five models are RoBERTa-Covid models, masked at 15, 25, 40, 60 and 80 percent both fully randomly and selectively. All models are trained using a learning rate of 3e-5, a batch size of 32, 10 warm-up steps, and an AdamW optimizer with an eps of 1e-6. Training occurs over 5 epochs with an early stopping mechanism. The two baseline models are the original BERT-large and RoBERTa-large models, trained only with random masking of 15% of tokens. We report random and selective F1 scores for all three categories (‘fake’, ‘true’ and ‘undetermined’ news) as well as the overall model F1 score. The scores are derived from 10-times repeated 10-fold cross-validation (CV). The standard deviation refers to the variance across CV runs. We use this approach because it is much more robust than a single CV pass, which may inflate or deflate results artificially. 10-times repeated 10-fold CV also helps evaluate the model’s ability to generalize to unseen data. This approach ensures that our averages result from a comprehensive understanding of the models’ ability to generalize to unseen data. Lastly, we do not report precision and recall scores in an effort to be concise and because the data are well-balanced, which results in similar precision and recall in all models. We report precision and recall scores in Appendix A.

**Table 1:** 10-times repeated 10-fold cross-validation performance matrix for different masking strategies, COVID data.

	Masking Strategy									
	Random F1 scores					Selective F1 scores				
% Masked	Fake	True	Undet.	Overall	$\bar{SD}$	Fake	True	Undet.	Overall	$\bar{SD}$
15%	0.848	${\text{0.811}}^{*}$	0.767	**0.809**	0.003	0.844	0.800	0.763	0.802	0.003
25%	0.847	0.797	0.767	0.804	0.004	0.848	${\text{0.805}}^{*}$	0.766	0.806	0.003
40%	**0.857**	0.800	0.767	0.808	0.004	**0.858**	0.803	**0.767**	**0.809**	0.005
60%	0.852	0.802	**0.770**	0.808	0.004	0.850	0.801	0.764	0.805	0.004
80%	0.848	0.796	0.767	0.805	0.004	0.844	0.793	0.758	0.799	0.004
BERT-large	0.782	0.772	0.724	0.760	0.003	-	-	-	-	-
RoBERTa-large	0.819	0.783	0.742	0.781	0.008	-	-	-	-	-

In fully random masking, we observe that *overall* performance differences (column 4) are small and not statistically significant –all are well within two standard deviations away from each other. This confirms the intuition that BERT and RoBERTa model creators had in using 15% for MLM. Differences are stark, however, in category-specific performance scores. Masking 40% of random tokens increases performance from 0.848 to 0.857 when detecting fake news, almost a full percentage point higher when compared to the default random masking of 15%. This comes at a cost, however, as true news performance decreases by 1.1 percentage points. Under the hood, the model has become better at predicting true positives (the fake news category is coded as 1), which has made it more precise. This is a positive development for a researcher building a fake news detector using this model. Note also that we only added 1GB worth of new unlabeled data to the base RoBERTa-large model, which is a drop in the ocean considering the 160GB the model was trained on. More data might improve the results further.

Selective masking further improves the results. The default model with 15% random *and* selective masking performs worse than its random masking counterpart. The 25% selective model improves fake news detection by 0.001 when compared to the random model, and 0.008 in true news detection. The latter result is statistically significant at the 0.05 level. The 40% model also improves on the random masking model, and does so by 0.001 and 0.003 in fake and true news. This result is not statistically significant but the improvement is consistent across all scores and is indicative that, with more training data, performance could improve further. As for models with 60 and 80 percent selective masking, both show a similar pattern of decreasing performance. The model with 60 percent selective masking loses 0.003 points on average, while the model with 80 percent selective masking loses 0.004 points in fake news detection, 0.003 in true news, and 0.006 in overall performance. This is in line with [13], who show decreasing performance in models with high rates of masking. It also shows that high levels of selective masking leave too few instances of the new word in the training data, making it more difficult for the model to learn.

In terms of substantive significance, while the gains are relatively small and they may not result in major changes in downstream analyses, there are two situations in which they are impactful. First is rare events data, where any gains in performance—even small ones—may have a significant impact on downstream analyses. Second, there may be cases where statistical significance is in the margins, and more accurate data may result in either stronger findings or in preventing type-I errors. Generally, even if gains appear small, performance improvements yield better data and, consequently, more robust and reliable results.

## Conclusion

This article shows that increasing the rate of random masking in RoBERTa models using social science texts can increase category-based performance. It also shows modest gains when using selective masking, a technique whereby we mask specific high-information tokens to increase learning. We illustrate the approach with the example of fake news stemming from the Coronavirus pandemic.

As we show throughout the paper, masking more than 15% of tokens improves category-specific performance. For our example, we find the optimal masking rate, as well as the optimal selective masking rate, is between 25 and 40 percent of the pretrained data. However, the exact rate of masking will vary depending on the task and domain, so we recommend researchers to test multiple masking rates to maximize category-specific performance. This is particularly important in situations where researchers have a preference between false positives and false negatives. Similarly, we show that *selective masking* can yield performance improvements in specialized texts where either new or highly specific tokens need to be added to the tokenizer.

While we have focused on the application of selective masking in data related to the social sciences, masking *select* high-information words can be of vital importance across other fields where domain-specific terms are recurrent and constantly being updated. Our example addresses COVID-19-related corpora and the classification of fake news, but a similar process can be useful to improve performance of models classifying academic literature on COVID-19 [39], or models trained to answer COVID-19 related questions [40]. Other domains, such as finance, legal studies, and biology, have shown the importance of domain-specific terms that are required in the pretraining data to improve performance (see [41]). In fact, we consider selective training and masking to be one of the key areas of future research for encoder-decoder models in the age of generative AI. In many highly specialized fields, increases in classifier performance may be best gained through the approach introduced in this article. This is especially relevant in academia, where many debates use highly specialized texts and language. Further research should continue to explore how selective masking improves performance in other applications.

Finally, we also stress how the category-specific gains from selective masking can be particularly relevant for rare-events data. In recent years, the use of machine-learning models to detect rare events has increased in political science [42], economy [43], criminology [44], and medicine [45], among others. For unbalanced training data, gains in hard-to-predict category can outweigh the losses from easier-to-predict categories. In rare-event data, increasing the accuracy of the prediction of the rare-event category is a sought-out goal [46]. As a more general conclusion, we show that different masking rates and masking strategies will affect downstream performance. The gains are not equal across categories and increases in one can be accompanied by decreases in another. It is ultimately incumbent upon the researcher to determine what is their goal when training models for prediction task, and adapt their pretraining and masking strategy accordingly. To this end, we provide an easy to implement, low cost approach to achieve this.

## Supporting information

AppendixA–D Appendices A through D include more information on precision and recall scores, the COVID-19 rumor dataset, results using DeBERTa, and an alternative test using a different dataset. (PDF)
