# Supplementary material for: Behind the mask: Random and selective masking in transformer models applied to specialized social science texts
Source: PLoS One. 2025 Feb 21;20(2):e0318421. doi: 10.1371/journal.pone.0318421 (PMC11844826; doi:10.1371/journal.pone.0318421)
Supplement: AppendixA–D — (PDF) [file pone.0318421.s001.pdf]

## Appendix for ‘Behind The Mask’


**A Precision and Recall Scores** **2**

**B The COVID-19 Rumor Dataset** **3**

**C DeBERTa results** **4**

**D Results with a Different Dataset: Racism in Ecuador** **5**

## Appendix A: Precision and Recall Scores

Tables A1 and A2 show the precision and recall scores, respectively, for all random and selective models in Table 1 (main article). The fact that we have slightly higher precision scores than recall scores for the fake news category indicates that the model has fewer false positives for fake rumors, which is more acceptable as this is the category that researchers are usually more concerned about. Conversely, the models have higher recall than precision scores for 'true' rumors, indicating a higher number of false positives for true news. Overall, however, the models tend to have very similar recall and precision scores (columns 4 and 8).

| | **Masking Strategy** | | | | | | | |
|---|---|---|---|---|---|---|---|---|
| | **Random Precision scores** | | | | **Selective Precision scores** | | | |
| **% Masked** | Fake | True | Undet. | Overall | Fake | True | Undet. | Overall |
| 15% | 0.871 | 0.782 | 0.794 | 0.810 | 0.877 | 0.784 | 0.775 | 0.803 |
| 25% | 0.873 | 0.779 | 0.781 | 0.805 | 0.884 | 0.783 | 0.775 | 0.807 |
| 40% | 0.876 | 0.779 | 0.791 | 0.809 | 0.877 | 0.780 | 0.785 | 0.781 |
| 60% | 0.876 | 0.788 | 0.779 | 0.810 | 0.868 | 0.782 | 0.785 | 0.807 |
| 80% | 0.879 | 0.769 | 0.792 | 0.806 | 0.874 | 0.776 | 0.768 | 0.799 |

**Table A.1:** Precision scores for random and selective models in Table 1.

| | **Masking Strategy** | | | | | | | |
|---|---|---|---|---|---|---|---|---|
| | **Random Recall scores** | | | | **Selective Recall scores** | | | |
| **% Masked** | Fake | True | Undet. | Overall | Fake | True | Undet. | Overall |
| 15% | 0.833 | 0.848 | 0.749 | 0.816 | 0.820 | 0.827 | 0.763 | 0.812 |
| 25% | 0.828 | 0.824 | 0.764 | 0.811 | 0.821 | 0.837 | 0.765 | 0.814 |
| 40% | 0.844 | 0.829 | 0.755 | 0.815 | 0.843 | 0.833 | 0.755 | 0.814 |
| 60% | 0.834 | 0.825 | 0.769 | 0.815 | 0.838 | 0.829 | 0.753 | 0.812 |
| 80% | 0.826 | 0.834 | 0.758 | 0.813 | 0.822 | 0.819 | 0.758 | 0.806 |

**Table A.2:** Recall scores for random and selective models in Table 1.

## Appendix B: The COVID-19 Rumor Dataset

The COVID-19 rumor dataset was collected by Cheng et al. (2021) to understand and "fight against rumors, conspiracy, misinformation, fake news, and disinformation." It can also be used for "sentiment analysis and other rumor classification tasks, including stance verification of COVID-19 rumors" (Cheng et al. 2021). The authors collected a total of 6,834 rumors around the COVID-19 pandemic from various news sources (4,129) and from Twitter (2,705) between January and March, 2020. The authors then refer to various authoritative fact-checking platforms such as poynter.org or factcheck.org to classify each text. For Twitter, the research team collected tweets using tags such as "COVID-19", "coronavirus", and "COVID" (Cheng et al. 2021, 2). For the news sources, the authors used *mitmproxy* (Cortesi et al., 2020), which is an open-source interactive HTTPS proxy that collected select news reports from Google browser.

One of the potential limitations of this data collection effort is in the lack of clarity around the sources for the news dataset. It is unclear what the exact set of sources is *mitmproxy* algorithm, which could potentially generate a disproportionate amount of fake news. It could also draw on fake news that are more distinctly fake, and less so on sources that produce less obvious, more nuanced fake news. Second, the rumors were collected only between January and March of 2020, an early phase of the coronavirus pandemic where much was still unknown about the new virus. While fact-checkers may have considered a given rumor to be false, it is no guarantee that the text was *intended* to be fake news at the time it was produced. Instead, its falseness could have been due to lack of knowledge about the virus. These drawbacks could lead to potential biases in the dataset and researchers should be aware of these issues when they use the dataset. For our purposes, however, these risks are mitigated because we consider the data to be internally valid and therefore performance measures should be reliable even in the face of these issues.

## Appendix C: DeBERTa results

| Model | Masking strategy | Avg. F1-scores | | | | $\overline{SD}$ |
|---|---|---|---|---|---|---|
| | | **Fake** | **True** | **Undet.** | **Overall** | |
| DeBERTa-large | Original (no FT) | 0.835 | 0.782 | 0.758 | 0.792 | 0.003 |
| | 40% random (FT) | 0.850 | 0.800 | 0.764 | 0.805 | 0.004 |
| | 40% random + 40% selective (FT) | 0.847 | 0.808 | 0.766 | 0.807 | 0.002 |

**Table C.1:** Results with DeBERTa-large models using the COVID-19 rumor dataset.
*Notes:* FT = Further trained on COVID-19 data. 'Original' model refers to Microsoft AI's original model with no further training. Both '40% random' and '40% random + 40% selective' models have been further trained with the COVID-19 data described in the main manuscript. All F1-score results are averages of 10-times repeated 10-fold cross-validation (100 models).

Table C1 shows the results extended to a selected sample of DeBERTa-large models. First is the original DeBERTa model without further training. The F1-score for detecting fake news is 0.835, and 0.782 for true news. Performance increases with a further trained model with 40% random masking, which in the main manuscript was one of the best performing models. The F1-score for fake news increases to 0.850 and for true news to 0.800, both of which are statistically meaningful changes (over two standard deviations). The third model adds selective masking of 40% of the coronavirus-related tokens, and yields improved performance in the category that generally yields lower F1-scores, true news. The F1-score for true news jumps to 0.808, again a statistically meaningful difference when compared to the second model. For fake news, the model underperforms slightly but the difference is not statistically meaningful. These results show that the findings in the main article extend to other encoder-decoder models using Masked Language Modeling (MLM) as the main learning mechanism.

## Appendix D: Results with a Different Dataset: Racism in Ecuador

Dávila et al. (2024) predict different forms of racism in large corpora in the Ecuadorian context. In their work, they further pre-train a XLM-RoBERTa model using domain specific data (e.g., Twitter data), manually adding tokens relevant to their training data. To further pre-train the model, the authors use an unlabeled set of over 1.7 million tweets collected between January 2018 and December 2020.[1] The tweets were searched using terms that were more likely to include racist language in Ecuador. They also add a list of 20 tokens that strongly signaled overt and covert forms of racism and shorthand expressions used to avoid being flagged as inappropriate content by Twitter. According to Dávila et al. (2024), these words were either not in the pretrained vocabulary or appeared in an unrelated context. The list of added tokens, as well as the tokens used for the initial embedding, are shown in Table D.1.[2]

**Table D.1:** Added Tokens

| **Added Tokens** | **Tokens Used for Initial Embedding** |
|---|---|
| guangudo | longo |
| huangudo | longo |
| cholo | longo |
| indiada | longo |
| emplumado | longo |
| plumífero | longo |
| rocoto | longo |
| bobolongo | longo |
| longanizo | longo |
| jíbaro | longo |
| mmv | f**ker, idiot, stupid |
| mmvs | f**ker, idiot, stupid |
| mmvgs | f**ker, idiot, stupid |
| mamaverga | f**ker, idiot, stupid |
| hp | f**ker, idiot, stupid |
| hijodeputa | f**ker, idiot, stupid |
| hdp | f**ker, idiot, stupid |
| hdlgp | f**ker, idiot, stupid |
| pndj | f**ker, idiot, stupid |
| pendejo | stupid, idiot |

To further explore the effects of selective masking, we use the same pretraining data and selectively mask the tokens they manually add. In Table D1, we show difference in performance

[1] The full pretraining dataset can be accessed at: `https://www.dropbox.com/scl/fi/2zjlgvxbtuemzwaqsa8as/pre_train_ecuador.txt?rlkey=jszx462etsoqq6ubbpbqk27q7&st=l84ni86i&dl=0` or by emailing the authors of this article.

[2] The full finetuning dataset can be accessed at: `https://www.dropbox.com/scl/fi/ywodhbrovpl8azsimjaeu/tm_07.xlsx?rlkey=d8187ir954hzksyhyuw7u2cg2&st=j06o5kti&dl=0` or by emailing the authors of this article.

from selectively masking these added tokens. Performance improvements are small but noticeable with covert racism with 20% random plus 40% selective as well as with 40% random plus 60% selective. Considering that the standard deviation is 0.002, the increase from 0.724 (40% random only) to 0.728 is statistically significant, as is the change from 0.723 (20% selective only) to 0.728. Moreover, the average F1 score for covert racism with any combination of random plus selective masking is 0.726, and only 0.718 and 0.717 for only random and only selective masking, respectively. As with the main paper, a combination of selective and random masking produces the best performance, especially with rates between 25 and 60 percent of masking.

| | F1 Scores | | | | |
|---|---|---|---|---|---|
| **Masking Strategy** | **No Racism** | **Overt Racism** | **Covert Racism** | **Overall** | $\overline{SD}$ |
| 15% Random | 0.860 | **0.695** | 0.723 | **0.760** | 0.0217 |
| 20% Random | **0.864** | 0.687 | 0.722 | 0.758 | 0.0247 |
| 40% Random | 0.861 | 0.685 | **0.724** | 0.757 | 0.0248 |
| 60% Random | 0.858 | 0.683 | 0.708 | 0.750 | 0.0366 |
| 80% Random | 0.859 | 0.683 | 0.712 | 0.751 | 0.0257 |
| 10% Selective | **0.864** | **0.696** | 0.722 | **0.760** | 0.0254 |
| 20% Selective | 0.862 | 0.689 | **0.723** | 0.758 | 0.0252 |
| 40% Selective | 0.859 | 0.683 | 0.716 | 0.753 | 0.0254 |
| 60% Selective | 0.860 | 0.685 | 0.710 | 0.752 | 0.0295 |
| 80% Selective | 0.859 | 0.684 | 0.706 | 0.750 | 0.0273 |
| 15% Random + 20% Selective | 0.863 | 0.688 | 0.725 | 0.758 | 0.0254 |
| 20% Random + 40% Selective | **0.864** | **0.693** | **0.728** | **0.762** | 0.0246 |
| 40% Random + 60% Selective | 0.862 | 0.689 | 0.728 | 0.760 | 0.0248 |
| 60% Random + 80% Selective | 0.862 | 0.689 | 0.721 | 0.757 | 0.0208 |

**Table D.2:** F1 scores for random and selective masking XLM-R with the Ecuador racism dataset.
